# *DNASE1L3* Mediates Hepatocellular Carcinoma Tumor Growth and Organoid Models via the Wnt/**β**-Catenin Signaling Pathway

**DOI:** 10.32604/or.2025.071739

**Published:** 2026-02-24

**Authors:** Shulong Zhang, Yijun Zhao, Li Geng, Feihong Song, Li Feng, Jun Jiang, Qianqian Cai, Fei Fan

**Affiliations:** 1 Department of General Surgery, Shanghai Xuhui Central Hospital, Zhongshan-Xuhui Hospital, Fudan University, Shanghai, 200031, China; 2 Department of the Third Ward of Special Treatment, Shanghai Eastern Hepatobiliary Surgery Hospital, Shanghai, 200438, China; 3 Endoscopy Center, Minhang Hospital, Fudan University, No. 170 Xinsong Road, Shanghai, 201199, China; 4 Shanghai Key Laboratory of Molecular Imaging, Jiading District Central Hospital Affiliated Shanghai University of Medicine and Health Sciences, Shanghai, 201318, China

**Keywords:** Hepatocellular carcinoma, *DNASE1L3;* Wnt/β-catenin signaling pathway, organoid models, tumor growth

## Abstract

**Background:**

Hepatocellular carcinoma (HCC) is a highly lethal malignancy driven by both intrinsic oncogenic pathways and immune microenvironmental regulation. Emerging evidence suggests that *DNASE1L3* may influence tumor biology and immune responses; however, its specific roles in HCC progression and macrophage-mediated regulation remain unclear. This study aimed to elucidate the biological functions of *DNASE1L3* in HCC and to determine how it modulates tumor behavior and immune interactions.

**Methods:**

Bioinformatics analyses of the GSE41804 and Cancer Genome Atlas-Liver Hepatocellular Carcinoma (TCGA-LIHC) datasets were used to identify hub genes. Functional assays assessed the impact of *DNASE1L3* on HCC cell proliferation, migration, invasion, and cell cycle progression. The effects of *DNASE1L3* on macrophage polarization and the Wnt/β-catenin signaling pathway were examined using a co-culture system. An HCC organoid model was established to further validate its regulatory function.

**Results:**

Eight prognostic signature genes were identified, with *deoxyribonuclease I-like 3 (DNase I-like 3)* selected as the hub gene. *DNASE1L3* overexpression suppressed HCC cell growth, inhibited migration and invasion, induced G1 arrest, and modulated epithelial-mesenchymal transition (EMT) markers. *DNASE1L3* knockdown promoted M2-like macrophage polarization. Mechanistically, *DNASE1L3* interacted with β-catenin to enhance its ubiquitination and degradation, thereby inhibiting Wnt/β-catenin signaling and reducing PD-L1 expression. *DNASE1L3* overexpression similarly restricted organoid growth and suppressed pathway activity.

**Conclusion:**

*DNASE1L3* acts as a negative regulator of HCC progression by targeting the Wnt/β-catenin pathway and reducing PD-L1 expression, thereby influencing both tumor cell behavior and macrophage-mediated immune responses.

## 1 Introduction

The most prevalent primary liver cancer is Hepatocellular carcinoma (HCC), which is mostly associated with chronic liver disease [1]. Although diagnostic and therapeutic approaches, such as surgical resection, liver transplantation, and targeted therapies, have made notable progress, patients with HCC still face poor prognoses characterized by frequent recurrence and metastasis [2]. Therefore, the goal of modern HCC research is to improve clinical outcomes while also elucidating the complex molecular pathways that initiate and advance tumors.

Immunotherapy strategies, such as checkpoint inhibitors and cellular therapies, have emerged for HCC treatment [3,4]. Recent studies indicate that the Wnt/β-catenin signaling pathway not only promotes tumor proliferation in HCC but also substantially remodels the tumor immune microenvironment. Specifically, β-catenin activation can suppress the infiltration of dendritic cells (DCs) and CD8^+^ T cells, thereby weakening antitumor immune responses [5,6]. Moreover, within tumor-associated macrophages (TAMs), Wnt/β-catenin signaling promotes M2-like polarization through c-Myc, enhancing their immunosuppressive functions and pro-tumor activity [7]. Importantly, studies have shown that Wnt pathway inhibitors, such as XAV939, can markedly downregulate *PD-L1* protein expression in HCC cells and enhance the efficacy of anti-*PD-L1* therapy [8]. This mechanism suggests that aberrant activation of the Wnt/β-catenin pathway may induce immune evasion by upregulating *PD-L1*, thereby reducing the effectiveness of immune checkpoint inhibitors.

*DNASE1L3*, located on chromosome 3, encodes human deoxyribonuclease I-like 3 (DNase I-like 3), an enzyme that degrades free DNA and is involved in apoptosis, immunological control, and cell signaling. In HCC, *DNASE1L3* has been identified as a prognostic gene, with its expression positively correlated with recurrence-free survival (RFS) [9]. Its downregulation, as demonstrated by Xiao et al., inhibits tumor progression via apoptosis induction and glycolysis suppression, associated with better prognosis [10]. The research of Wang et al. further validates the role of *DNASE1L3*, indicating its downregulation in HCC tissues predicts favorable survival post-resection [11]. These findings highlight the critical role of *DNASE1L3* in HCC progression and its potential as a prognostic biomarker. Mechanistic studies have demonstrated that *DNASE1L3* directly interacts with components of the β-catenin destruction complex, such as GSK-3β and AXIN, thereby promoting β-catenin ubiquitination and degradation. This interaction suppresses β-catenin nuclear translocation and downregulates its downstream targets, including c-Myc, p21, and p27, ultimately restraining Wnt/β-catenin–mediated cell cycle progression and EMT in HCC cells [12]. Moreover, *DNASE1L3* expression is closely associated with the tumor immune microenvironment. Analyses using TIMER and clinical HCC samples reveal positive correlations between *DNASE1L3* levels and the infiltration of DCs, CD8^+^ T cells, and macrophages [13]. Together, these findings suggest that DNASE1L3 not only restrains tumor cell proliferation and EMT via Wnt/β-catenin inhibition but also may modulate the immune microenvironment, highlighting its potential as both a prognostic marker and a therapeutic target in HCC.

Despite promising techniques like CAR-T cell treatment and anti-PD-1/PD-L1 antibodies, there are still obstacles to overcome in the field of HCC immunotherapy [14]. Patient-derived organoids (PDOs) offer a preclinical model that preserves tumor heterogeneity and microenvironmental features, enabling the study of tumor progression and drug response [15]. Co-culturing liver PDOs with cancer-associated fibroblasts (CAFs) has been shown to promote proliferation and enhance resistance to therapies such as sorafenib, regorafenib, and 5-fluorouracil [16]. This synergistic interaction underscores the complex nature of the tumor microenvironment in fostering drug resistance. Liver organoids have been shown to better model drug resistance compared with patient-derived xenografts [17]. Taken together, these findings highlight the utility of liver organoids in improving HCC treatment strategies and improving treatment outcomes.

HCC, characterized by high mortality and frequent recurrence, remains a substantial challenge despite developments in diagnostic and therapeutic approaches. The complexity of the HCC tumor microenvironment and modulation of the immune response are key areas for improving treatment outcomes. This study aims to further delineate the molecular mechanisms underlying HCC progression, with a particular focus on the role of *DNASE1L3*, a gene previously linked to various cancers but understudied in the context of liver cancer. Recent findings on the immunological landscape of HCC highlight the possibility of immune checkpoint inhibitors and the modulation of tumor microenvironments using innovative preclinical models such as organoids. These models have showed potential in recreating tumor cellular heterogeneity and microenvironment, allowing for more precise assessment of treatment responses.

Our research intends to explore the impact of *DNASE1L3* manipulation within such models, assessing its influence on cellular proliferation, immune evasion, and response to therapy. Specifically, this study will investigate the impact of *DNASE1L3* overexpression and knockdown on the Wnt/β-catenin signaling pathway and PD-L1 level in HCC cells and organoids. By integrating cell and organoid models, this study aimed to discover new therapeutic targets and enhance comprehension of the function of *DNASE1L3* in the immune environment of HCC.

## 2 Materials and Methods

### 2.1 Analysis of Differential Gene Expression in Public Databases

The GSE41804 dataset (20 HCC, and 20 adjacent normal liver tissues), obtained from the Gene Expression Omnibus (GEO; https://www.ncbi.nlm.nih.gov/gds/) database. Additionally, we analyzed data from the Clinical Bioinformatics Assistant platform (https://www.aclbi.com/static/index.html#), which includes the TCGA-LIHC dataset containing 371 HCC and 50 normal samples. After normalization of the expression data, we performed comparative analysis using the Limma R package (version 3.56.2). Genes with |log_2_FC| > 1 were considered differentially expressed, where log_2_FC > 1 indicated upregulation and log_2_FC < −1 indicated downregulation, with statistical significance defined as a false discovery rate (FDR) < 0.05.

### 2.2 Weighted Gene Co-Expression Network Analysis (WGCNA)

We analyzed the differentially expressed genes from GSE41804 through WGCNA implementation in R (version 4.3.2). Prior to analysis, missing values were removed, and low-expression genes were filtered out to ensure data quality. Network construction employed a soft-threshold power of β = 14 to ensure scale-free topology. The choice of β was determined using the pickSoftThreshold function in the WGCNA R package (version 1.73), which evaluates a range of candidate powers based on the scale-free topology fit index (R^2^) and mean connectivity. β = 14 was selected as it was the lowest power at which the scale-free topology fit index first exceeded 0.85 while maintaining sufficient mean connectivity, ensuring that the constructed network approximates a scale-free topology, a key assumption of WGCNA. Gene connections were quantified using a topological overlap matrix (TOM), followed by hierarchical clustering to identify distinct modules. Each module, represented as a dendrogram branch with unique coloring, contained genes grouped based on their expression patterns and weighted correlation values. The biological significance of these modules was evaluated through correlation with clinical parameters.

### 2.3 Batch Survival Analysis of Overlapping Genes

We utilized the online Venn diagram analysis tool (https://bioinformatics.psb.ugent.be/webtools/Venn/) to identify overlapping genes among three groups: upregulated DEGs, downregulated DEGs, and genes in the turquoise module. Following that, batch survival analysis was done on 306 elevated overlapping genes using R software to investigate their influence on the overall survival (OS) prognosis of HCC patients. The prognostic significance was evaluated through univariate Cox regression, yielding hazard ratios (HR) and corresponding *p*-values with 95% confidence intervals (CIs). Among these, 106 genes showed significant prognostic differences. Finally, a nomogram was created to visually represent the top 20 genes having prognostic importance, which were selected based on having the most significant *p*-values in the survival analysis.

### 2.4 Analysis of Prognostic Gene Signatures in the LIHC Risk Model

106 genes with prognostic potential were analyzed by Least Absolute Shrinkage and Selection Operator (LASSO) Cox regression using the glmnet R package (version 4.1–2). Model parameters were optimized by ten-fold cross validation to determine the minimum criterion λ (λ.min = 0.0606) for selecting predictive signatures. The risk score for each patient was calculated using the following formula: Riskscore = (−0.0151)*CPEB3 + (−0.0186)*CYP2C9 + (−0.185)*NTF3 + (−0.0224)*SPP2 + (−0.0123)*LCAT + (−0.0002)*GGT5 + (−0.0719)*DNASE1L3 + (−0.0429)*CFHR3. The TCGA-LIHC cohort was separated into high- and low-risk groups based on expression patterns. Survival differences between groups were evaluated using Kaplan-Meier curves and log-rank tests in R (version 4.3.2), and median survival times and HR were calculated for risk assessment. Prediction accuracy was quantified using time-dependent receiver operating characteristic (timeROC) R package (version 0.4) to generate receiver operating characteristic (ROC) curves and calculate AUC values for 1-, 3-, and 5-year survival predictions, with higher values indicating stronger prognostic ability.

### 2.5 Expression Analysis of Eight Characteristic Genes

Following the identification of eight significant genes from the prognostic risk model, we analyzed their expression levels in the GSE41804 and TCGA-LIHC datasets. The Sangerbox website (http://vip.sangerbox.com/home.html) was used for data processing and box plot visualization to analyze their expression in the tumor and normal groups. A non-parametric test (rank-sum test) was applied for two-group comparisons, and *p*-values < 0.05 were considered statistically significant.

### 2.6 Clinical Specimens

Patients at Shanghai Eastern Hepatobiliary Surgery Hospital, The Third Affiliated Hospital of Naval Medical University, provided a set of five liver cancer specimens and matching adjacent non-tumor liver tissues for this investigation after surgery, and informed agreement was obtained. Each HCC case was histopathologically confirmed by a qualified histopathologist to ensure diagnostic accuracy and consistency between samples. This study was approved by the Ethics Committee of the Third Affiliated Hospital of Second Military Medical University (Approval No.: BHBY2022-K020-B001). The study was conducted in accordance with the Declaration of Helsinki. Written informed consent was obtained from all participants, and institutional consent was obtained from Shanghai Eastern Hepatobiliary Surgery Hospital, The Third Affiliated Hospital of Naval Medical University.

### 2.7 Cell Lines and Culture

HepaRG cells (YS2323C) were purchased from Shanghai Yaji Biotechnology (Shanghai, China), while liver cancer cell lines (HCCLM3, TCHu270; HepG2, SCSP-510; Huh7, SCSP-526; Hep3B, SCSP-5045) and THP-1 (SCSP-567) monocytes were obtained from the Cell Bank of the Chinese Academy of Sciences (Shanghai, China). All cell lines were authenticated using short tandem repeat (STR) profiling and confirmed to be free of mycoplasma contamination. HepaRG and HCC lines were maintained in DMEM (11965092, Gibco, Thermo Fisher Scientific, Waltham, MA, USA), while THP-1 cells were grown in RPMI 1640 medium (11875085, Gibco). Media were supplemented with 10% fetal bovine serum (FBS, A5669701, Gibco) and 1% penicillin-streptomycin (P1400, Solarbio, Beijing, China). Cells were incubated at 37°C with 5% CO_2_.

### 2.8 Differentiation of THP-1 Macrophages and Co-Culture of HCC Cell Lines

To establish an *in vitro* co-culture system, THP-1 monocytes (1 × 10^6^ cells per well) were differentiated into macrophages using 10–100 nm Phorbol 12-myristate 13-acetate (PMA, 16561-29-8, Sigma, St. Louis, MO, USA) for 24–48 h, which promotes cell adherence and macrophage phenotype formation by activating the protein kinase C (PKC) signaling pathway. The resulting cells were polarized into M1 (baseline, pro-inflammatory) and M2 (anti-inflammatory) macrophages following established protocols [18]. Following differentiation and polarization, the THP-1 macrophages were cocultured with HepG2 and Huh7 cell lines. For co-culture experiments, a 24-mm Transwell system (3450, 0.4 μm pore polycarbonate membrane, Corning, Corning, NY, USA) was used. HCC cells (5 × 10^5^ cells per well) were seeded in the lower chamber, while differentiated macrophages (1 × 10^5^ cells per well) were placed in the upper chamber. Both cell types were pre-cultured separately for 24 h in complete medium. The assembled system was maintained at 37°C with 5% CO_2_ for 12 h, after which cells were collected for subsequent analyses, including quantitative real-time polymerase chain reaction (qRT-PCR) and Western blotting (WB) to assess gene and protein expression related to macrophage–HCC interactions. All experiments were performed in at least three independent biological replicates, with technical replicates included as appropriate.

### 2.9 Transient Transfection for Gene Expression

For transfection studies, HCC cells (HepG2 and Huh7) were seeded at 2 × 10^5^ cells per well in 24-well plates. The overexpression plasmid used to modulate *DNASE1L3* expression was constructed using the pcDNA3.1 vector (pcDNA3.1, V79020, Invitrogen, Carlsbad, CA, USA), while *DNASE1L3* knockdown was achieved using specific siRNAs (si-*DNASE1L3*-1 and si-*DNASE1L3*-2). The empty pcDNA3.1 vector served as the negative control for overexpression, and a non-targeting siRNA (si-NC) was used as the negative control for knockdown. Plasmid DNA (500 ng per well) or siRNAs were diluted in Opti-MEM and mixed with Lipofectamine 3000 (L3000150, Invitrogen) according to the manufacturer’s recommended ratios. The mixture was incubated at room temperature for 15–30 min to allow nucleic acid–lipid complex formation, followed by incubation with cells for 4–6 h at 37°C and 5% CO_2_. The medium was then replaced with complete growth medium, and cells were cultured for an additional 24 h to allow sufficient overexpression or knockdown. The final siRNA concentration was 80 nm. The siRNA sequences were as follows: si-*DNASE1L3*-1 (sense: GCUUGGAAGAAACACAUAUAA; antisense: UUAUAUGUGUUUCUUCCAAGC), si-*DNASE1L3*-2 (sense: GACAUCAUACUCGUGAUGGAA; antisense: UUCCAUCACGAGUAUGAUGUC), and si-NC (sense: UUUUCCGAACGUGUCACGUTT; antisense: ACGUGACACGUUCGGAAAATT).

### 2.10 Cell Treatment

To examine proteasome activity and Wnt signaling in HCC, HepG2 and Huh7 cells were treated with MG132 (S2619, 20 μg/mL, Selleck Chemicals, Houston, TX, USA) or XAV939 (S1180, 10 μm, Selleck Chemicals) for 8 and 10 h, respectively. In addition, ICG-001 (S2662, 10 μm, Selleck Chemicals) was used to target the Wnt/β-catenin pathway for 24 h. All inhibitors were dissolved in dimethyl sulfoxide (DMSO), and the final solvent concentration was kept consistent across experimental groups. The treatment was applied to *DNASE1L3* knockdown HepG2 cells and co-cultured with PMA-differentiated THP-1 macrophages at a 5:1 ratio (HCC cells: macrophages) for 24 h. All experiments were performed with at least three independent biological replicates and appropriate technical replicates.

### 2.11 HCC Organoid Culture

PDOs were cultured from HCC tissue samples. The tissue was minced and enzymatically digested using Liberase TM (0.25 mg/mL, 5401119001, Sigma) and DNase I (0.1 mg/mL, AMPD1, Sigma) at 37°C until a homogeneous cell suspension was obtained. The suspension was then filtered and mixed with Advanced DMEM/F12 containing 10% FBS. Following a brief centrifugation (300× *g*, 5 min, 4°C), the cell pellet was resuspended in cold PBS after the supernatant was extracted. Cell clusters were suspended in a solubilized basement membrane and placed in 24-well plates. The cultivation medium consisted of Advanced DMEM/F12 added to with B-27 (2%, 17504044, Gibco), N-2 (1%, 17502048, Gibco), GlutaMAX (2 mm, 35050061, Gibco), HEPES (10 mm, 15630-080, Gibco), N-acetyl-L-cysteine (1 mm, A7250, Sigma), nicotinamide (10 mm, N0636, Sigma), and several growth factors including gastrin (10 nm, G9145, Sigma), EGF (50 ng/mL, 236-EG, R&D Systems, Minneapolis, MN, USA), FGF10 (100 ng/mL, 345-FG, R&D Systems), HGF (50 ng/mL, 294-HG, R&D Systems), forskolin (5 μm, F6886, Sigma), A8301 (500 nm, SML0788, Sigma), Y27632 (10 μm, Y0503, Sigma), and dexamethasone (100 nm, D4902, Sigma). The organoids were passaged weekly upon reaching confluence. Matrigel was then displaced, collected, and dissolved Matrigel and released the organoids by incubating for one hour at 4°C with a cell recovery solution (354253, Corning). The organoids were subsequently digested with TrypLE solution (12604021, Gibco) for 5–10 min at 37°C, resuspended in Matrigel, and replated to form new organoids. Organoid morphology and growth characteristics were evaluated using brightfield imaging with an Olympus CK30 microscope (Tokyo, Japan) and captured with cellSens Standard software (Olympus, Japan). Images were subsequently processed and analyzed using ImageJ (version 1.53, NIH, USA). Organoids were routinely passaged every 7–10 days using TrypLE Express, and experiments were conducted using organoids at passages 2–5 to ensure consistency. Each organoid line was derived from an independent patient sample and experiments were performed in triplicate to confirm reproducibility.

### 2.12 qRT-PCR

Following the manufacturer’s instructions, total RNA was extracted from THP-1 monocytes, HCC cells, and THP-1-derived M0, M1, and M2 macrophages using TRIzol reagent (15596018, Invitrogen). cDNA was synthesized using PrimeScript RT (RR047A, Takara, Kusatsu, Shiga, Japan) in a 20 μl reaction volume with 500 ng RNA input per reaction, incubated at 37°C for 15 min, followed by 85°C for 5 s. Using the SYBR Green PCR Master Mix (RR820A, Takara), qRT-PCR was carried out in a 20 μl reaction volume on a StepOnePlus Real-Time PCR System (4376600, Applied Biosystems, Thermo Fisher Scientific). Thermal cycling conditions were: 95°C for 30 s, followed by 40 cycles of 95°C for 5 s and 60°C for 30 s. The levels of gene expression were adjusted to GAPDH. All target levels were determined using the 2^−ΔΔCT^ method. Table 1 had a collection of primer sequences.

**Table 1:** Primer sequences for qRT-PCR

Target	Direction	Sequence (5^′^-3^′^)
*DNASE1L3*	Forward	TGCTCCTTCAACGTCAGGTC
*DNASE1L3*	Reverse	CCAGCTTTTCCCTGTTCAGC
*Wnt1*	Forward	TGTGGAAATGAGGTTGGGGG
*Wnt1*	Reverse	CGTGGCTCTGTATCCACGTT
*Wnt2*	Forward	TGGACAACCGCTACATGACC
*Wnt2*	Reverse	AGGGGGCTTCCGTTGAGATA
*Wnt2b*	Forward	AAAAGGGGCCAGGAGGATTC
*Wnt2b*	Reverse	TGTTTTCCTCCCAGAGTGCT
*Wnt3*	Forward	CACAACACGAGGACGGAGAA
*Wnt3*	Reverse	GCTTCCCATGAGACTTCGCT
*Wnt3a*	Forward	TTTGGTGGGATGGTGTCTCG
*Wnt3a*	Reverse	ACCAGCATGTCTTCACCTCG
*Wnt4*	Forward	TCGTCTTCGCCGTCTTCTCA
*Wnt4*	Reverse	ATGACTTCCAGGTTCCGCTT
*Wnt5a*	Forward	TCTGGCTCCACTTGTTGCTC
*Wnt5a*	Reverse	CGACCACCAAGAATTGGCTTC
*Wnt5b*	Forward	CAAGAGAGCGAGAAGACTGGA
*Wnt5b*	Reverse	AATGACCACCAGGAGTTGGC
*Wnt6*	Forward	CGAGAGTGCCAGTTCCAGTT
*Wnt6*	Reverse	ATAGAACAGGCCTGCGTGAC
*Wnt7a*	Forward	GGACTATGAACCGGAAAGCG
*Wnt7a*	Reverse	TCCTATGACGATGATGGCGTC
*Wnt7b*	Forward	CACCATGCTTCTACTGTCGC
*Wnt7b*	Reverse	GGCTAGGCCAGGAATCTTGTT
*Wnt8a*	Forward	GTGCCTACAGAACAGCCACA
*Wnt8a*	Reverse	GGTTGGACATGGTCACATGC
*Wnt8b*	Forward	AAGGAGAAGTACCACGCAGC
*Wnt8b*	Reverse	GCGTTTTGTTCTCCAGGCAG
*Wnt9a*	Forward	ATGACTCGCCTAGCTTCTGC
*Wnt9a*	Reverse	CACTCCACATAGCAGCACCA
*Wnt9b*	Forward	TCTTGCCATAGCAGGCTTCC
*Wnt9b*	Reverse	GCAGCCAGGGATTCTGAGAG
*Wnt10a*	Forward	TCCTGTTCTTCCTACTGCTGC
*Wnt10a*	Reverse	GGATGCCCTGTATGGCTGAG
*Wnt10b*	Forward	ACTAGTGAAGCCCAGGCAAC
*Wnt10b*	Reverse	TTAGAGCCCGACTGCACAAC
*Wnt11*	Forward	TCAGAATGTTCTGCGGGACC
*Wnt11*	Reverse	GAGAAGTGCCACCCCAAAGA
*Wnt16*	Forward	CATGCAGCTCACCACTTGC
*Wnt16*	Reverse	GCAGGTACGGTTTCCTCTTG
*NOS2*	Forward	CTGCAAGCACAATGGGGAGT
*NOS2*	Reverse	CGTCGGTAGAGAGACTGCTG
*CXCL10*	Forward	TCATCCCTGCGAGCCTAT
*CXCL10*	Reverse	CTTGATGGTCTTAGATTCCGGAT
*CXCL9*	Forward	CAAATCCCTCAAAGACCTCAAAC
*CXCL9*	Reverse	GATCTCCGTTCTTCAGTGTAGC
*TNF-α*	Forward	TCACTGGAGCCTCGAATGTC
*TNF-α*	Reverse	TCTGTGAGAAGGCTGTGCA
*CD206*	Forward	CACCATCGAGGAATTGGACT
*CD206*	Reverse	ACAATTCGTCATTTGGTCA
*CD163*	Forward	ATTCATCATCCTCGGACCCAT
*CD163*	Reverse	CCCAGCACAACGACCACCT
*ARG1*	Forward	CCACATCGCTCAGACACCAT
*ARG1*	Reverse	GGCAACAATATCCACTTTACCAGAGT
*IL10*	Forward	GATGCCTTCAGCAGAGTGAA
*IL10*	Reverse	GCAACCCAGGTAACCCTTAAA
*TGF-β1*	Forward	GGCCAGATCCTGTCCAAGC
*TGF-β1*	Reverse	GTGGGTTTCCACCATTAGCAC
*Fzd4*	Forward	CCTCGGCTACAACGTGACC
*Fzd4*	Reverse	TGCACATTGGCACATAAACAGA
*Fzd7*	Forward	GTGCCAACGGCCTGATGTA
*Fzd7*	Reverse	AGGTGAGAACGGTAAAGAGCG
*Fzd9*	Forward	AGTTTCCTCCTGACCGGTTT
*Fzd9*	Reverse	GTGGCAGCAGTACATGGTTG
*β-catenin*	Forward	GTTGAGCACCTGTTTGCCTG
*β-catenin*	Reverse	GGCTGTCAGGTTTGATCCCA
*Axin2*	Forward	TATCCAGTGATGCGCTGACG
*Axin2*	Reverse	CGGTGGGTTCTCGGGAAATG
*c-Myc*	Forward	GCCCCTAGTGCTGCATGAG
*c-Myc*	Reverse	CCACAGACACCACATCAATTTCTT
*PD-L1*	Forward	GGAGCCATCTTATTATGCCTT
*PD-L1*	Reverse	TCACTTTGCTTCTTTGAGTTTGT
*GAPDH*	Forward	TGACAACAGCCTCAAGATCG
*GAPDH*	Reverse	GTCTTCTGGGTGGCAGTGAT

### 2.13 Nuclear-Cytoplasmic Fractionation Assay

The protein levels of DNASE1L3 and β-catenin in the cytoplasm and nucleus of HepG2 cells (approximately 5 × 10^6^ cells per sample) overexpressing DNASE1L3 were measured by cellular fractionation using a Nuclear and Cytoplasmic Protein Extraction Kit (P0027, Beyotime, Shanghai, China) and a protease inhibitor (CW2200S, CoWin Biosciences, Nanjing, China). Following the manufacturer’s protocol, cells were lysed in 200 μl cytoplasmic extraction buffer and vortexed for 15 s, followed by incubation on ice. The cytoplasmic fraction was obtained after centrifugation at 16,000× *g* (4°C, 10 min). The remaining pellet underwent nuclear protein extraction with similar centrifugation conditions. DNASE1L3 (1:1000, 67041-1-Ig, Wuhan Sanying, Wuhan, China), β-catenin (1:5000, ab32572, Abcam, Cambridge, UK), H3 (1:1000, ab1791, Abcam), and GAPDH (1:1000, ab8245, Abcam) protein levels were measured in both the cytoplasmic and nuclear fractions using WB assay.

### 2.14 Wb Assay

Protease and phosphatase inhibitors (CW2200S, CoWin Biosciences) were added to RIPA buffer (R0010, Solarbio) before cells and tissues were lysed. Protein concentrations were determined using the Bicinchoninic Acid (BCA) assay (P0012, Beyotime). Equal amounts of protein (20 μg per lane) were separated by SDS-PAGE (10% gel for most targets; 12% gel for low-molecular-weight proteins) and transferred onto PVDF membranes. The membranes were blocked with 5% non-fat milk in TBST (Tris-buffered saline with 0.1% Tween 20) for 1 h at room temperature and incubated with primary antibodies overnight at 4°C. The primary antibodies were as follows: DNASE1L3 (1:1000, 67041-1-Ig, Wuhan Sanying), E-cadherin (1:1000, ab40772), vimentin (1:1000, ab92547), N-cadherin (1:5000, ab76011), c-Myc (1:1000, ab32072), P21 (1:1000, ab109520), P27 (1:5000, ab32034), CD206 (1:1000, ab300621), ARG1 (1:1000, ab133543), β-catenin (1:5000, ab32572), PD-L1 (1:1000, ab228415), and Axin2 (1:1000, ab109307), H3 (1:1000, ab1791), Cyclin D1 (10,000, ab134175), with GAPDH (1:1000, ab8245) used as the internal control. All antibodies, except for DNASE1L3, were purchased from Abcam. After washing three times with TBST, membranes were incubated with species-specific HRP-conjugated secondary antibodies (anti-mouse IgG, 1:10000, ab6728, Abcam; anti-rabbit IgG, 1:5000, ab6721, Abcam) for 1 h at room temperature. Protein bands were identified using enhanced chemiluminescence (ECL) detection reagents (P0018AS, Beyotime) and recorded using the ChemiDoc System (1708280, Bio-Rad, Hercules, CA, USA) following incubation with secondary antibodies. All primary antibodies were validated by the manufacturers for specificity via knockout/knockdown controls or by referencing previous publications. Secondary antibodies were species-specific and verified for minimal cross-reactivity.

### 2.15 Cell Counting Kit-8 (CCK-8) Assay

The CCK-8 test was used to evaluate the cells’ vitality (KGA1606-1000, KeyGEN, Nanjing, China). In 96-well plates, HCC cells were cultivated at a density of 5 × 10^3^ cells per well in a total culture volume of 100 µL per well. After overexpression of *DNASE1L3*, 10 µL of was added to each well, and cells were incubated for 2 h at 37°C. Absorbance at 450 nm was measured using a microplate reader (ST-960, Kehua Technologies, Inc., Shanghai, China), with values from blank wells subtracted. Measurements were taken at 450 nm at one, two, three, and four days post-treatment. Experiments were performed with at least three biological replicates to ensure reproducibility.

### 2.16 Transwell Assay

Transwell tests were used to assess the cells’ capacity for migration and invasion. HCC cells were resuspended in serum-free media to produce a single-cell suspension for the migration test. The lower chamber of the Transwell system (3422, Corning) received 500 μL of complete medium containing 10% FBS, whereas the top chamber received 200 μL of the cell suspension, which contained around 5 × 10^4^ cells. For the invasion assay, the upper surface of the membrane was pre-coated with Matrigel (356234, Corning) at a concentration of 1 mg/mL and a coating thickness of ~100 μm to simulate the extracellular matrix. The cells were maintained in a humidified incubator at 37°C for twenty-four hours. A cotton swab was used to remove the non-migrated cells from the top chamber, and DAPI (C1005, Beyotime) at 1 μg/mL was used to stain the migrated cells on the lower surface for 15 min at room temperature. Following staining, the membrane was washed with PBS and air-dried. Cell migration was quantified by counting five random fields at ×200 magnification using an Olympus CKX53 microscope (Olympus, Tokyo, Japan), with representative images captured. For the invasion assay, a layer of Matrigel was pre-coated onto the membrane of the Transwell insert to simulate the extracellular matrix. The remaining procedures were identical to the migration assay. Invasive cells were quantified using the same counting method.

### 2.17 Flow Cytometry Analysis

HCC cells were planted (1 × 10^5^ cells per well) on 6-well plates and cultivated for 24 h. After 48-h treatment, cells were trypsinized (0.25%), centrifuged (300× *g*, 3 min, 4°C), and treated in 100 μL of with RNase A (100 μg/mL, 30 min). Following overnight fixation in 70% alcohol at 4°C, cells were stained with 50 μL of propidium iodide (PI, 50 μg/mL, 30 min). Analysis was performed using a flow cytometer (CyFlow Cube8, Jiyuan, Guangzhou, China) equipped with a 488 nm laser and detection in the FL2 channel. Data were analyzed with FlowJo software (version 10.8.1, Becton Dickinson, USA).

### 2.18 Co-Immunoprecipitation (Co-IP)

Total protein was extracted from HepG2 cells according to manufacturer directions, and the protein content was determined using a protein quantification assay. Five milligrams of total protein were used as the starting material for immunoprecipitation. Specific antibodies were used to incubate protein samples against the target proteins at 4°C. Depending on the experimental requirements, antibodies such as β-catenin (1:30, ab32572), GSK-3β (1:30, ab280376), Axin2 (1:150, ab109307), CK1 α (1:30, ab206652), APC (1:70, ab40778), or anti-Flag (1:30, ab205606) were added. Target proteins were immunoprecipitated with 4 μg specific antibody or IgG control using Protein A/G beads overnight at 4°C. The eluted complexes were analyzed by WB to assess protein interactions.

### 2.19 Sample Preparation and Histological Staining

HCC PDO was preserved in 10% neutral buffered formalin and 4% paraformaldehyde, then sequentially dehydrated in graded ethanol (70%, 80%, 95%, and 100%, 5 min each), cleared in xylene, and paraffin embedded. Sectioning was performed to obtain 4 µm thick tissue sections and set up on glass slides. Hematoxylin and eosin (H&E) staining was performed using the H&E Staining Kit (C0105, Beyotime) to assess histopathological features under a Nikon Eclipse E200 light microscopy (Nikon, Japan).

### 2.20 Immunohistochemistry (IHC)

For tissue staining, antigen retrieval was performed in citrate buffer (pH 6.0) by heating in a microwave at 95°C for 15 min. Endogenous peroxidase activity was blocked with 0.3% NaBH4 (CAS:16940-66-2, Sinopharm Chemical Reagent, Shanghai, China) at room temperature for 10 min. The tissue sections were then processed using the Vectastain Elite ABC Kit (PK-6200, Vector Laboratories, USA) according to the manufacturer’s instructions. Primary antibody incubation was conducted overnight at 4°C using DNASE1L3 (1:200, 67041-1-Ig, Wuhan Sanying). Secondary antibody (anti-mouse IgG, 1:10000, ab205719, Abcam) was applied at room temperature for 1 h. Nuclei were visualized with hematoxylin counterstain (H8070, Solarbio) and immunoreactivity with DAB staining solution (DA1010, Solarbio) for 5 min. The growth of HCC organoids overexpressing DNASE1L3 was monitored daily using a Nikon Eclipse Ti-S phase-contrast microscopy (Nikon, Japan). Organoid size and morphology were assessed to analyze the impacts of overexpression DNASE1L3 on HCC organoid growth dynamics.

### 2.21 Statistical Analysis

R software was used for data analysis, with results expressed as mean ± SD (n = 3). Group comparisons employed ANOVA with Tukey’s test. Survival outcomes were evaluated using log-rank tests, and Cox regression identified prognostic indicators. Significance was defined at *p* < 0.05.

## 3 Results

### 3.1 Differential Gene Screening and Key Module Identification

**Figure 1:**
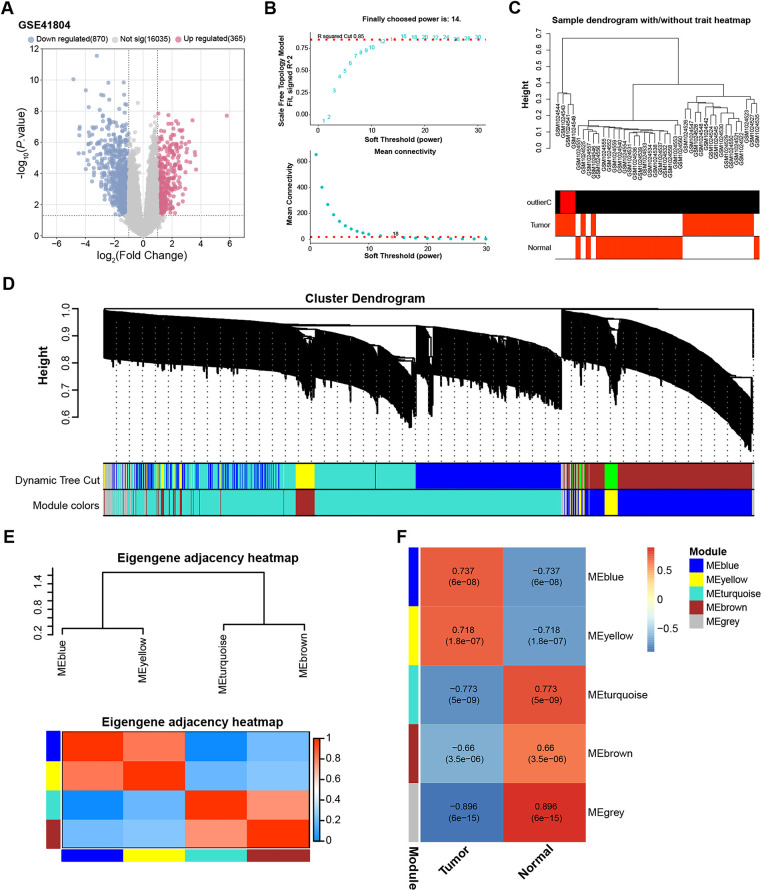
DEG identification and WGCNA in the GSE41804 dataset. (**A**) Volcano plot showing differentially expressed genes (DEGs) between HCC (n = 20) and non-tumor liver tissues (n = 20) in the GSE41804 dataset. |Log_2_ Fold Change| > 1 was used as the threshold to define DEGs. Genes with log_2_FC > 1 and *p* < 0.05 were defined as upregulated (365 genes, shown in red), whereas genes with log_2_FC < −1 and *p* < 0.05 were defined as downregulated (870 genes, shown in blue). (**B**) The upper figure shows the determination of the optimal soft threshold in the gene co-expression network, and the lower figure shows the mean connectivity for different soft thresholds. (**C**) Sample dendrogram and trait heat map, different branches represent different GSE41804 dataset samples. (**D**) Gene dendrogram obtained by average linkage hierarchical clustering. The colored rows below the dendrogram show module assignments determined by dynamic tree cuts. (**E**) Eigengene adjacency heat map of the correlation between module eigengenes and sample traits. (**F**) Heatmap of the correlation between gene modules and GSE41804 samples, the numbers in the modules represent the correlation coefficients and *p*-values. DEGs, differentially expressed genes

Transcriptome data of HCC were obtained from the GSE41804 dataset, which includes 20 HCC tissue samples and 20 matched adjacent normal liver tissues. Using this dataset, we first identified 1235 DEGs, comprising 365 upregulated and 870 downregulated genes (Fig. 1A). We then performed WGCNA on the GSE41804 dataset. As shown in Fig. 1B, the ideal soft threshold power to ensure scale-free topological model fitting was determined to be 14, R^2^ = 0.85 without scale. Afterwards, we conducted a thorough analysis of the clustering of 40 samples in the GSE41804 dataset (Fig. 1C). The WGCNA method then divides the genes into many modules, each of which is represented by a different hue, based on the patterns of gene co-expression in different samples (Fig. 1D,E). We examined the adjacencies of signature genes to evaluate the relationships between the identified modules. There was a significant association between the sample and the turquoise module, as indicated by the correlation coefficient of 0.773 (Fig. 1F). In summary, the analysis of differentially expressed genes and co-expression modules laid the foundation for identifying potential key genes in this study.

### 3.2 Gene Overlap Assessment and Survival Correlation

**Figure 2:**
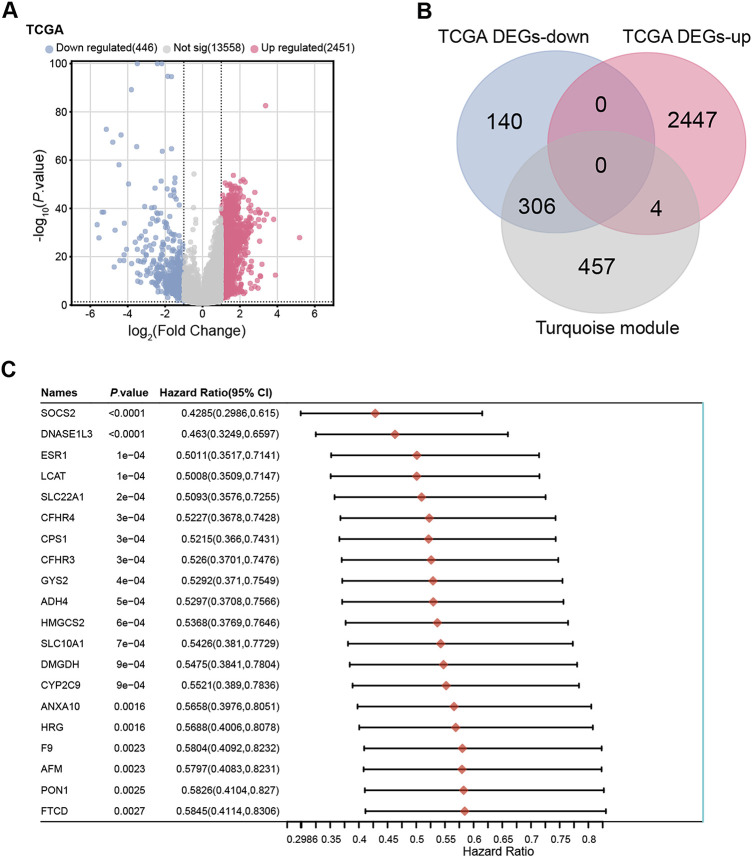
Identification and prognostic analysis of overlapping genes. (**A**) Volcano plot showing DEGs between HCC tissues (n = 371) and normal liver tissues (n = 50) in the TCGA-LIHC dataset (total n = 421). DEG was defined as |Log_2_ Fold Change|> 1 and *p* < 0.05, identifying 2451 upregulated genes (red) and 446 downregulated genes (blue). (**B**) The Venn diagram showed that 306 overlapping genes were identified from the down-regulated DEGs and turquoise modules of TCGA. (**C**) Prognostic analysis was performed on 306 overlapping genes, showing the top 20 genes with significant prognosis. TCGA, The Cancer Genome Atlas; LIHC, liver hepatocellular carcinoma; DEGs, differentially expressed genes

To validate and prioritize candidate genes, transcriptome data from the TCGA-LIHC dataset, which includes 371 HCC samples and 50 normal liver tissues, were analyzed. Differential expression analysis identified 2451 upregulated and 446 downregulated genes (Fig. 2A). Cross-over analysis by a bioinformatics platform revealed 306 overlapping genes between the turquoise module and down-regulated DEGs of TCGA (Fig. 2B). A batch survival analysis highlighted 106 overlapping genes with significant prognostic value, and a forest plot was utilized to depict the top 20 genes with their corresponding *p*-values and hazard ratios (Fig. 2C). Through survival analysis and overlap assessment, candidate genes with significant prognostic value were identified, providing a basis for further key gene selection.

### 3.3 Identification of Eight Signature Prognostic Genes

**Figure 3:**
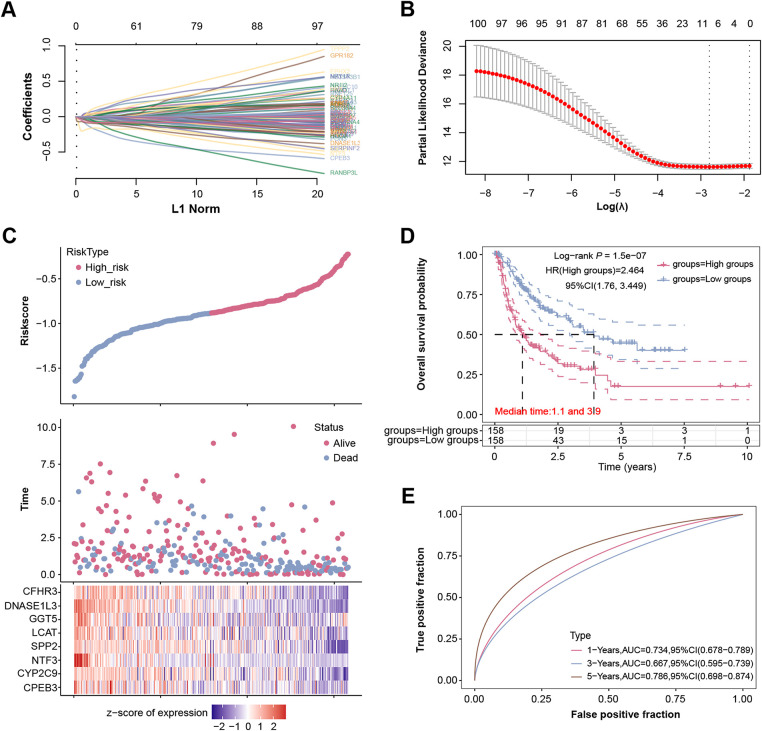
Identification of eight signature prognostic genes. (**A**) LASSO-Cox regression model analysis of 106 prognostically significant genes among 306 overlapping genes. (**B**) The relationship between 10-fold cross-validation partial likelihood deviation and log(λ). (**C**) Risk model analysis of the selected sample data, the upper panel shows the risk samples, the middle panel shows the survival status, and the lower panel shows the heatmap of the clustering distribution of signature genes. (**D**) The KM survival curve analysis of the two groups in the risk model, the red line indicates the high-risk group and the blue line indicates the low-risk group. (**E**) ROC curve analysis on the risk model in patients at 1, 3, and 5 years, the horizontal coordinate is a false positive fraction, and the vertical coordinate is a true positive fraction. LASSO, least absolute shrinkage, and selection operator; KM, Kaplan-Meier; ROC, receiver operating characteristic

Prognostic analysis was conducted on the 306 overlapping genes, revealing 106 genes with significant prognostic value. LASSO Cox regression analysis (λ = 0.0606) identified 8 signature genes (Fig. 3A,B). Risk score was calculated as: Riskscore = (−0.0151)*CPEB3 + (−0.0186)*CYP2C9 + (−0.185)*NTF3 + (−0.0224)*SPP2 + (−0.0123)*LCAT + (−0.0002)*GGT5 + (−0.0719)*DNASE1L3 + (−0.0429)*CFHR3. The high-risk group had lower survival rates than low-risk individuals, with median survival durations of 1.1 and 3.9 years, respectively (HR = 2.464; Fig. 3C,D). The model demonstrated strong predictive performance with a five-year AUC of 0.786 (Fig. 3E), highlighting these genes’ prognostic significance. By integrating immune-related analyses, candidate genes closely associated with the tumor microenvironment and macrophage polarization were further prioritized, laying the groundwork for subsequent studies on *DNASE1L3*.

### 3.4 Downregulation of Eight Significant Prognostic Genes in HCC

**Figure 4:**
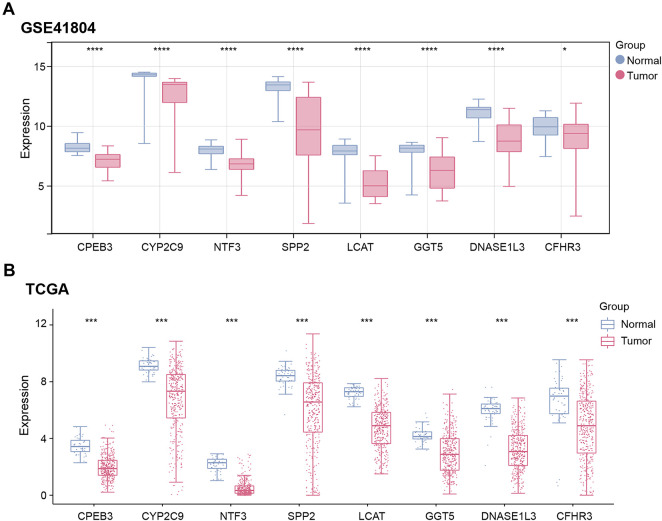
Identification and functional analysis of hub genes. (**A**,**B**) Expression of eight signature prognostic genes in tumor and control samples of (**A**) GSE41804 and (**B**) TCGA-LIHC datasets. Red represents the case group and blue represents the control group. TCGA, The Cancer Genome Atlas; LIHC, liver hepatocellular carcinoma. *p < 0.05, ***p < 0.001, ****p < 0.0001

Expression analysis revealed significant downregulation of the eight genes (*CPEB3*, *CYP2C9*, *NTF3*, *SPP2*, *LCAT*, *GGT5*, *DNASE1L3*, *CFHR3*) in tumor samples from both the GSE41804 dataset and the TCGA-LIHC dataset (Fig. 4A,B). This downregulation may implicate these genes in crucial metabolic and regulatory pathways that are altered during hepatocarcinogenesis. Notably, existing research has demonstrated that *DNASE1L3* can suppress glycolysis in hepatocellular cancer cells, promoting the tricarboxylic acid cycle involving the pathways of CEBPβ-p53-PFK1 and PTPN2-HK2, thereby inhibiting the progression of HCC [12]. Based on these integrative analyses and functional relevance, DNASE1L3 was selected as the key gene for further experimental investigation.

### 3.5 Overexpression of DNASE1L3 Inhibits HCC Cell Phenotypes

**Figure 5:**
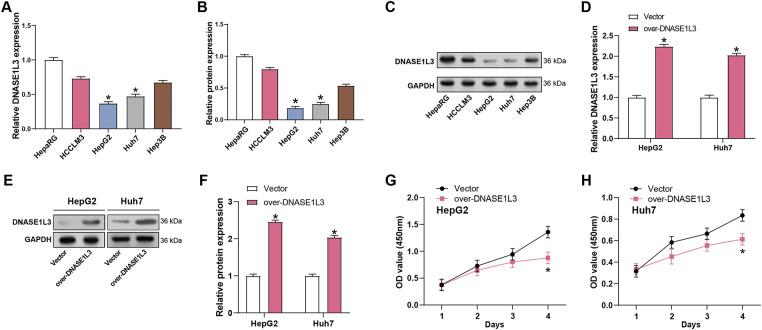

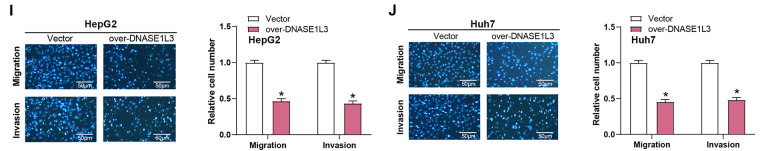
Overexpression of *DNASE1L3* inhibits HCC cell viability, invasion, and metastasis. (**A**) qRT-PCR detection of *DNASE1L3* mRNA levels in normal control cells (HepaRG) and HCC cell lines (HCCLM3, HepG2, Huh7, and Hep3B). (**B**) WB detection of *DNASE1L3* protein levels in normal control cells (HepaRG) and HCC cell lines (HCCLM3, HepG2, Huh7, and Hep3B). (**C**) Quantification of *DNASE1L3* protein expression relative to GAPDH in (**B**). (**D**) Relative expression of *DNASE1L3* mRNA in HepG2 and Huh7 cells transfected with *DNASE1L3* overexpression vector or control vector. (**E**) WB analysis of *DNASE1L3* protein expression in transfected HepG2 and Huh7 cells. GAPDH was used as a loading control. (**F**) Quantification of *DNASE1L3* protein expression relative to GAPDH in (**E**). (**G**,**H**) CCK-8 detects the effect of overexpression of *DNASE1L3* on cell viability in (**G**) HepG2 and (**H**) Huh7 cells. (**I**,**J**) Transwell was used to detect the effect of overexpression of *DNASE1L3* on cell migration and invasion in (**I**) HepG2 and (**J**) Huh7 cells. Scale bar: 50 μm. qRT-PCR, Quantitative real-time polymerase chain reaction; WB, Western blot; CCK-8, Cell counting kit-8. *p < 0.05

In HepG2 and Huh7 cells, *DNASE1L3* expression was substantially lower than in normal controls (Fig. 5A–C). Subsequent qRT-PCR and WB analyses demonstrated efficient overexpression of *DNASE1L3* (Fig. 5D–F). After successful overexpression validation (Fig. 5D–F), CCK-8 assays showed reduced cell viability in *DNASE1L3*-overexpressing HepG2 and Huh7 cells (Fig. 5G,H). Transwell assays further revealed that overexpression of *DNASE1L3* attenuated the invasive and migrating properties of HCC cells (Fig. 5I,J).

### 3.6 DNASE1L3 Modulates Cell Cycle and EMT Markers in HCC

**Figure 6:**
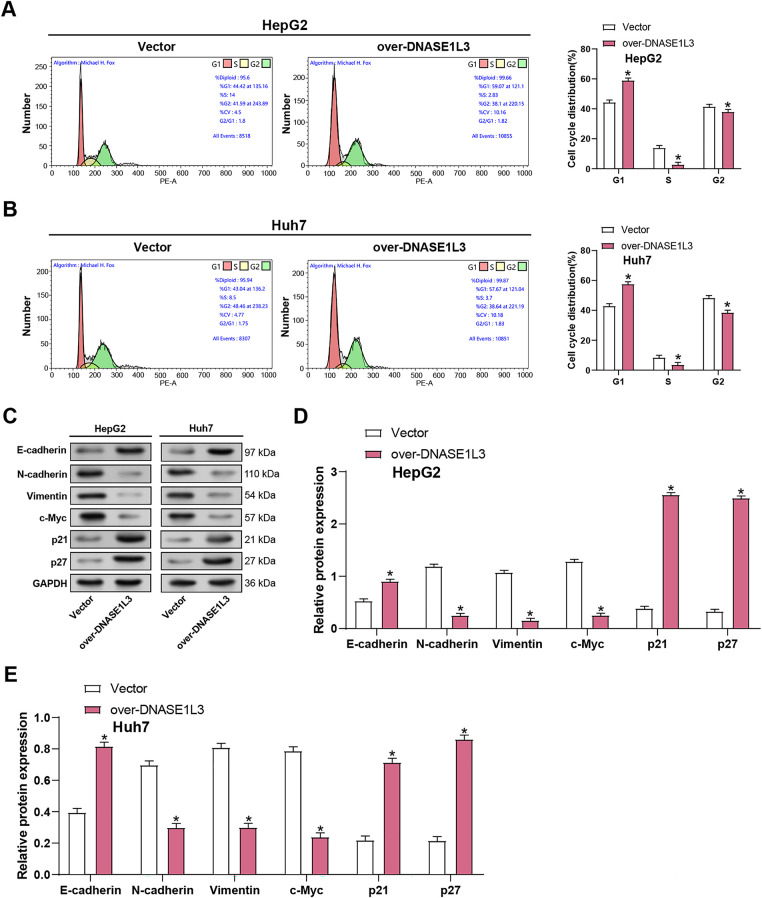
Overexpression of *DNASE1L3* causes cell cycle arrest in the G1 phase. (**A**,**B**) Cell cycle analysis of HepG2 (**A**) and Huh7 (**B**) cells transfected with *DNASE1L3* overexpression vector or control vector. Representative flow cytometry plots (left) and quantification of cell cycle distribution (right) are shown. (**C**) WB analysis of EMT markers (E-cadherin, N-cadherin, Vimentin), c-Myc, and cell cycle inhibitors (p21, p27) in HepG2 and Huh7 cells transfected with *DNASE1L3* overexpression vector or control vector. GAPDH serves as a loading control. (**D**,**E**) Quantification of protein expression levels relative to GAPDH in HepG2 (**D**) and Huh7 (**E**) cells. *p < 0.05

Flow cytometry revealed that overexpressed *DNASE1L3* induced G1 phase arrest (Fig. 6A,B). Concurrently, WB analysis observed a downregulation of EMT markers, including a decrease in c-Myc, N-cadherin, and vimentin levels, coupled with an upregulation of the epithelial marker E-cadherin (Fig. 6C–E). Additionally, the WB analysis indicated a rise in the expression of the G1 phase-associated proteins P21 and P27, suggesting a potential mechanism by which *DNASE1L3* overexpression influences cell cycle progression and EMT processes in the cellular context.

### 3.7 Knockdown of DNASE1L3 Promotes Macrophage Infiltration and M2-Like Polarization in HCC

**Figure 7:**
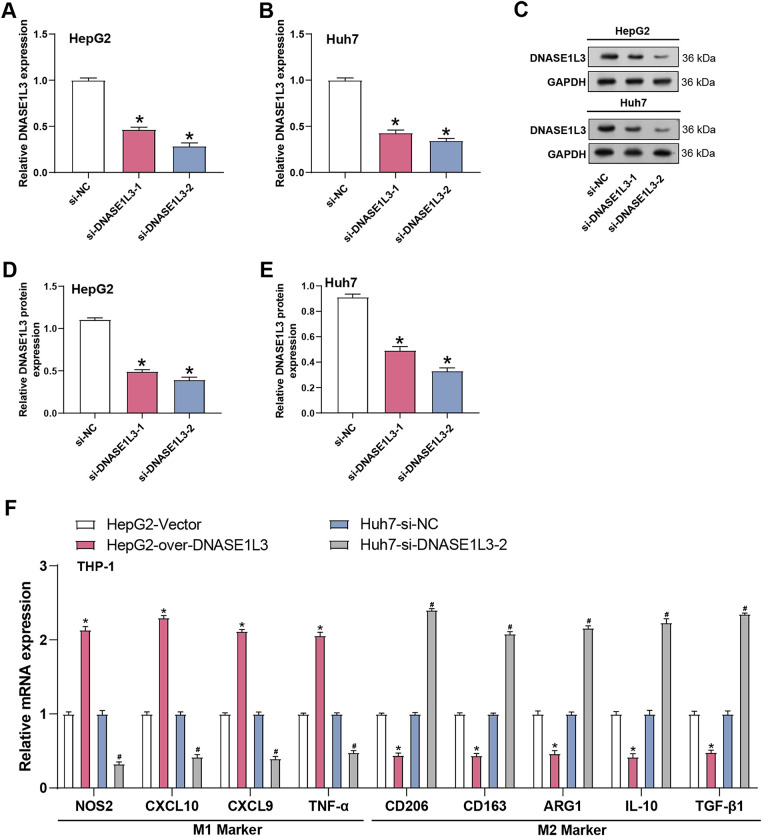
Knockdown of *DNASE1L3* promotes tumor infiltration and M2-like polarization of macrophages in HCC. (**A**,**B**) Relative *DNASE1L3* mRNA expression in HepG2 (**A**) and Huh7 (**B**) cells transfected with siRNA targeting DNASE1L3 (si-DNASE1L3-1 and si-DNASE1L3-2) or negative control (si-NC). *p < 0.05. (**C**) WB analysis of DNASE1L3 protein expression in HepG2 and Huh7 cells transfected with si-NC, si-*DNASE1L3*-1, or si-*DNASE1L3*-2. GAPDH serves as a loading control. (**D**,**E**) Quantification of DNASE1L3 protein expression relative to GAPDH in HepG2 (**D**) and Huh7 (**E**) cells. *p < 0.05. (**F**) Relative mRNA expression of M1 (*NOS2*, *CXCL10*, *CXCL9*, *TNF-α*) and M2 (*CD206*, *CD163*, *ARG1*, *IL-10*, *TGF-β1*) macrophage polarization markers in THP-1 cells co-cultured with HepG2 cells overexpressing *DNASE1L3* or Huh7 cells with *DNASE1L3* knockdown. *p < 0.05 vs. HepG2-Vector groups. ^#^*p* < 0.05 vs. Huh7-si-NC. HCC, hepatocellular carcinoma

Although *DNASE1L3* has traditionally been recognized for its role in extracellular DNA clearance, emerging evidence suggests that it also participates in immune regulation and inflammatory processes [19]. Together with the substantial signaling alterations we observed in HCC cells, we hypothesized that *DNASE1L3* may not only modulate the intrinsic biological behaviors of tumor cells but also influence the tumor immune microenvironment by regulating tumor-derived secretory factors-particularly those capable of shaping macrophage polarization. To test this hypothesis, we further employed a co-culture system to assess the impact of *DNASE1L3* dysregulation on macrophage phenotypes. *DNASE1L3* silencing efficiency was confirmed in HepG2 and Huh7 cells using two siRNAs, with si-*DNASE1L3*-2 showing greater effectivenes (Fig. 7A–E). To further understand the influence of *DNASE1L3* on macrophages, we created a co-culture system that monitored HCC cell-macrophage interactions. The levels of M1 markers (*NOS2*, *CXCL10*, *CXCL9*, and *TNF-α*) and M2 markers (*CD206*, *ARG1*, *CD163*, *IL10*, and *TGF-β1*) were assessed by co-culturing THP-1 differentiated macrophages with *DNASE1L3* knockdown/overexpression HCC cells. According to the findings, overexpression of *DNASE1L3* markedly increased M1 marker expression while decreasing M2 marker expression. On the other hand, induction with si-*DNASE1L3-*2 had the opposite effect, leading to a notable rise in M2 marker expression and a fall in M1 marker levels (Fig. 7F). According to these findings, *DNASE1L3* controls the polarization of macrophages in hepatocellular carcinoma by encouraging M1 polarization when overexpressed and M2 polarization when downregulated.

### 3.8 Differential Expression of Wnt Receptors and Activation of Wnt/***β***-Catenin Signaling Pathway in Polarized Macrophages

**Figure 8:**
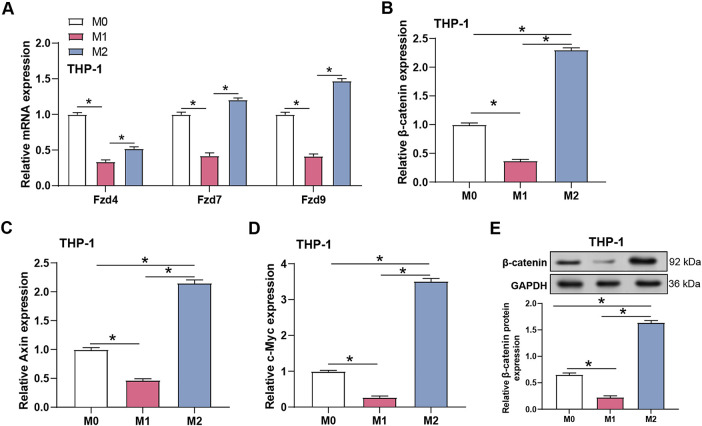
Differential expression of Wnt receptors and activation of Wnt/β-catenin signaling pathway in polarized macrophages. (**A**) qRT-PCR detection of the relative mRNA expression of Frizzled receptors (*Fzd4*, *Fzd7*, *Fzd9*) in THP-1 cells polarized to M0, M1, and M2 phenotypes. (**B**) qRT-PCR detection of the relative mRNA expression of *β-catenin* in THP-1 cells polarized to M0, M1, and M2. (**C**) qRT-PCR detection of the relative mRNA expression of *Axin2* in THP-1 cells polarized to M0, M1, and M2. (**D**) qRT-PCR detection of the relative mRNA expression of *c-Myc* in THP-1 cells polarized to M0, M1, and M2. (**E**) WB analysis (up) and quantification (down) of β-catenin protein expression in THP-1 cells polarized to M0, M1, and M2. GAPDH was used as a loading control. qRT-PCR, Quantitative real-time polymerase chain reaction. *p < 0.05

To determine whether the Wnt/β-catenin pathway participates in macrophage polarization, the mRNA expression levels of Wnt receptors and downstream signaling genes were analyzed in M0, M1, and M2 THP-1 macrophages using qRT-PCR (Fig. 8A). M1 macrophages showed consistently low expression of *Fzd4*, *Fzd7*, and *Fzd9*, whereas M2 macrophages displayed markedly elevated levels of these receptors, suggesting a higher responsiveness to Wnt signals. Consistently, β-catenin, Axin2, and c-Myc mRNA levels were significantly increased in M2 macrophages compared with M0 and M1 cells (Fig. 8B–D), and Western blotting confirmed substantially higher β-catenin protein levels in M2 macrophages (Fig. 8E). When combined, these results suggest that Wnt/β-catenin signaling is significantly active in M2 macrophages and may play a role in monocyte differentiation.

### 3.9 Wnt Ligands Released by HCC Cells Paracrinely Activate Wnt/***β***-Catenin Signaling in M2 Macrophages

**Figure 9:**
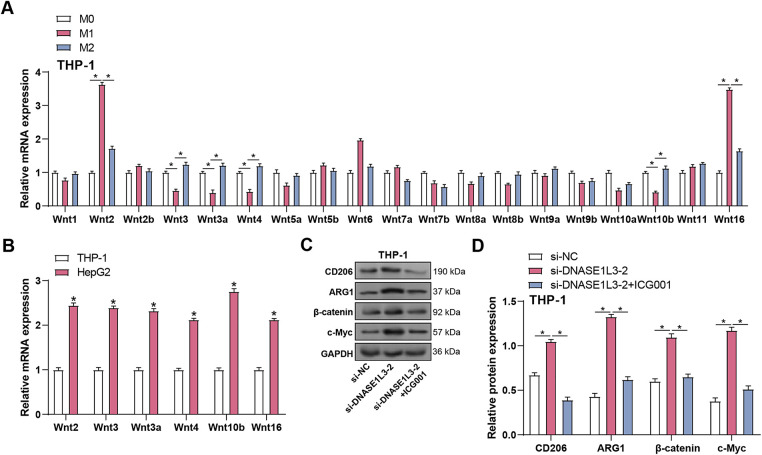
Wnt ligands secreted by HCC cells initiate Wnt/β-catenin signaling activation in M2 macrophages in a paracrine manner. (**A**) HCC cells with knockdown *DNASE1L3* were co-cultured with PMA-differentiated THP-1 macrophages. The expression of Wnt ligands among M0, M1, and M2 macrophages was determined by qRT-PCR. (**B**) HepG2 cells with knockdown *DNASE1L3* were co-cultured with PMA-differentiated THP-1 macrophages. qRT-PCR was used to compare the mRNA levels of *Wnt2*, *Wnt3*, *Wnt3a*, *Wnt4*, *Wnt10b*, and *Wnt16* in HepG2 cells with knockdown *DNASE1L3* and THP-1 cells after co-culture. (**C**) HepG2 cells with knockdown *DNASE1L3* were co-cultured with PMA-differentiated THP-1 macrophages. The cells were treated with or without ICG001 for 24 h. The protein levels of M2 macrophage markers (CD206, ARG1) and Wnt/β-catenin pathway components (β-catenin, c-Myc) were detected by WB. GAPDH was used as a loading control. (**D**) Quantification of protein expression levels by WB analysis in (**C**). qRT-PCR, Quantitative real-time polymerase chain reaction. *p < 0.05

Wnt ligands produced from macrophages have been demonstrated in several studies to stimulate Wnt signaling in tumor cells [20,21]. Through canonical Wnt/β-catenin signaling, tumor cell-derived Wnt ligands induce M2-like polarization of TAMs, which results in tumor growth, migration, metastasis, as well as immunosuppression in HCC [22,23]. The function of *DNASE1L3* in Wnt/β-catenin signaling and macrophage polarization was investigated in HCC. Distinct Wnt ligand patterns emerged across macrophage subtypes (Fig. 9A), with M1 macrophages showing elevated *Wnt2* and *Wnt16* expression, while M2 macrophages exhibited higher *Wnt3*, *Wnt3a*, and *Wnt4* levels compared to other subtypes. However, comparison with HepG2 cells revealed that HCC cells expressed significantly higher levels of *Wnt2*, *Wnt3*, *Wnt3a*, *Wnt4*, *Wnt10b*, and *Wnt16* (Fig. 9B), indicating that HCC cells may serve as the major paracrine source of Wnt ligands in the tumor microenvironment. We knocked down *DNASE1L3* in HepG2 cells using siRNA and co-cultured them with THP-1 macrophages to study its influence on Wnt/β-catenin signaling. Silencing of *DNASE1L3* led to up-regulation of the expression of M2 macrophage markers (CD206 and ARG1) and Wnt/β-catenin pathway proteins (β-catenin and c-Myc) in THP-1 cells according to WB analysis. Treatment with ICG001, a Wnt/β-catenin inhibitor, reversed these effects, demonstrating that *DNASE1L3* controls macrophage polarization via the Wnt/β-catenin signaling pathway (Fig. 9C,D). These results demonstrate that *DNASE1L3* restrains tumor-derived Wnt/β-catenin signaling and inhibits the M2-like polarization of macrophages.

### 3.10 DNASE1L3-***β***-Catenin Interaction Mechanism

**Figure 10:**
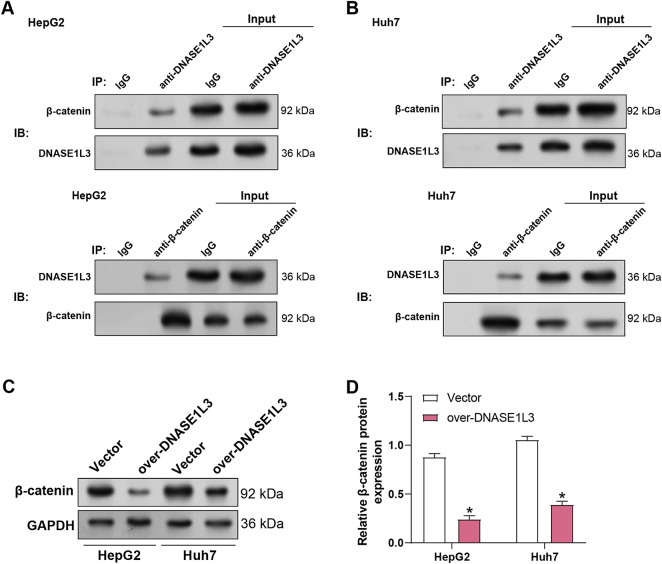
DNASE1L3 binds to β-catenin and inhibits its activation. (**A**,**B**) Co-IP assays showing interaction between DNASE1L3 and β-catenin in HepG2 (**A**) and Huh7 (**B**) cells. IgG serves as a negative control. (**C**) WB analysis of β-catenin expression in HepG2 and Huh7 cells transfected with *DNASE1L3* overexpression vector or control vector. GAPDH serves as a loading control. (**D**) Quantification of β-catenin protein expression relative to GAPDH in HepG2 and Huh7 cells from (**C**). Co-IP, Co-immunoprecipitation. *p < 0.05

According to our study, *DNASE1L3* knockdown encourages M2-like polarization of macrophages in HCC, maybe by triggering the macrophages’ Wnt/β-catenin signaling pathway. Therefore, we sought to investigate whether *DNASE1L3* is associated with the Wnt/β-catenin signaling pathway and enhances HCC cell proliferation. To this end, we performed co-IP assays in HepG2 and Huh7 cell lines (Fig. 10A,B). The results showed that DNASE1L3 and β-catenin physically interact in both cell lines. Furthermore, overexpression of *DNASE1L3* in HCC cells led to a significant reduction in β-catenin protein levels, as determined by WB analysis (Fig. 10C,D). These results demonstrate that DNASE1L3 physically interacts with β-catenin.

### 3.11 DNASE1L3 Recruits the ***β***-Catenin Degradation Complex to Promote ***β***-Catenin Ubiquitination and Degradation and Inhibit Its Nuclear Translocation

**Figure 11:**
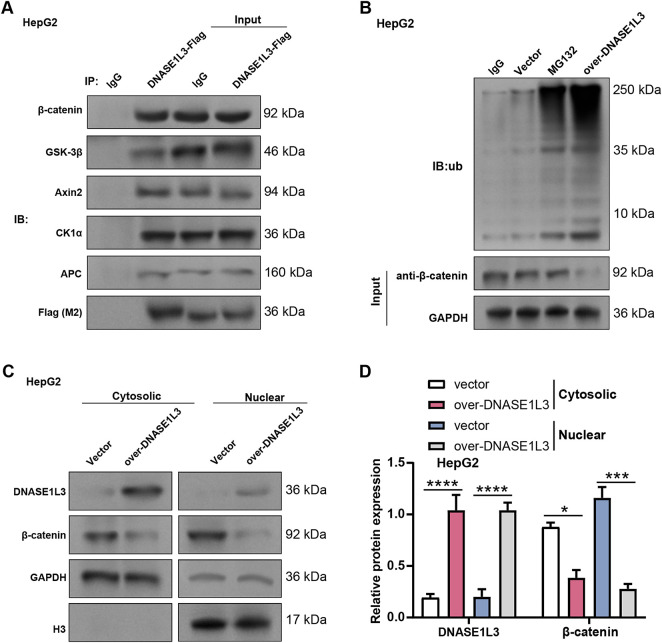
*DNASE1L3* recruits the β-catenin degradation complex to promote β-catenin ubiquitination and degradation and inhibit its nuclear translocation. (**A**) Interaction of DNASE1L3 with the β-catenin degradation complex (GSK-3β, Axin2, CK1 α, APC, and β-catenin) in HepG2 cells was determined by Co-IP with exogenous (Flag-DNASE1L3). IgG was used as a negative control. (**B**) Ubiquitination of β-catenin protein in vector or over-DNASE1L3 cells. Immunoprecipitation was performed with β-catenin antibody, followed by immunoblotting with ubiquitin antibody. MG132-treated vector cells served as a positive control. (**C**) The levels of DNASE1L3 and β-catenin proteins in the cytoplasm and nucleus of overexpressed Hep3B cells were detected by nuclear-cytoplasmic fractionation. GAPDH and histone H3 were used as cytoplasmic and nuclear markers, respectively. Co-IP, Co-immunoprecipitation. (**D**) Protein expression levels in the cytoplasm and nucleus of overexpressed Hep3B cells were quantified by WB analysis under the conditions described in (**C**). *p < 0.05; ***p < 0.001; ****p < 0.0001

*DNASE1L3* has been shown to bind to *β-catenin* and prevent its activation. We hypothesized that *DNASE1L3* might attract additional β-catenin destruction complexes to interact with β-catenin protein and subsequently control the protein level of β-catenin in order to further validate this regulatory mechanism. Co-IP assays in HepG2 cells showed that *DNASE1L3* interacted with key components of β-catenin and its degradation complex (Fig. 11A). Analysis of β-catenin stability demonstrated that treatment with MG132 markedly increased the accumulation of ubiquitinated β-catenin. Notably, compared with the vector control, *DNASE1L3* overexpression further enhanced the ubiquitination of β-catenin (Fig. 11B), indicating that *DNASE1L3* promotes β-catenin ubiquitination in HepG2 cells. Subcellular fractionation experiments in HepG2 cells showed that *DNASE1L3* overexpression reduced β-catenin levels in the cytoplasmic and nuclear fractions (Fig. 11C,D). This reduction was more obvious in the nuclear fraction, indicating that *DNASE1L3* may preferentially target nuclear β-catenin for degradation or inhibit its nuclear translocation. These findings indicate that *DNASE1L3* interacts with the β-catenin degradation complex, increases β-catenin ubiquitination, and lowers β-catenin levels in the cytoplasm and nucleus.

### 3.12 DNASE1L3 Modulates PD-L1 through Wnt/***β***-Catenin Signaling

**Figure 12:**
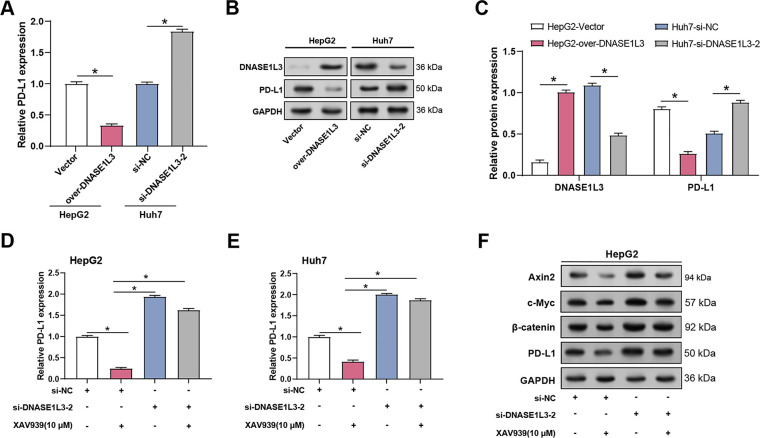

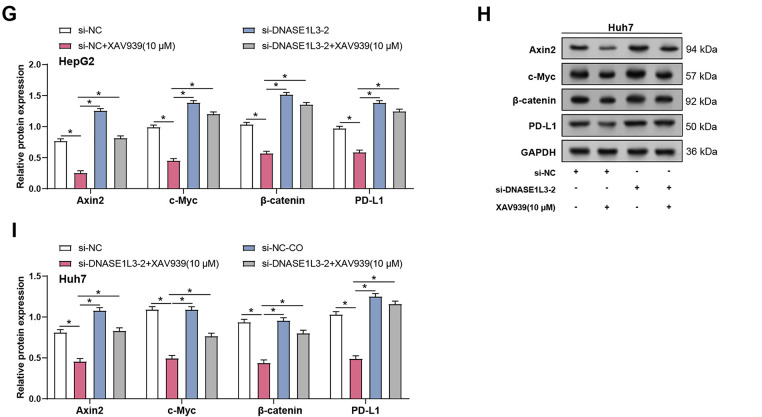
DNASE1L3 knockdown-induced M2-like macrophages upregulate PD-L1 levels in HCC cells. (**A**) qRT-PCR detection of the relative expression of *PD-L1* mRNA in HepG2 and Huh7 cells with *DNASE1L3* overexpression or knockdown. (**B**) WB analysis of DNASE1L3 and PD-L1 protein expression in HepG2 and Huh7 cells with *DNASE1L3* overexpression or knockdown. GAPDH was used as a loading control. (**C**) Quantitative analysis of DNASE1L3 and PD-L1 protein expression (**B**). (**D**,**E**) qRT-PCR detection of the relative expression of *PD-L1* mRNA in HepG2 (**D**) and Huh7 (**E**) cells transfected with si-NC or si-*DNASE1L3*-2, with or without XAV939 treatment. (**F**,**H**) WB analysis of Wnt/β-catenin pathway components (Axin2, c-Myc, β-catenin) and PD-L1 in HepG2 (**F**) and Huh7 (**H**) cells transfected with si-NC or si-*DNASE1L3*-2, with or without XAV939 treatment. GAPDH served as a loading control. (**G**,**I**) Quantification of protein expression levels by WB analysis in HepG2 (**G**) and Huh7 (**I**) cells. HCC, hepatocellular carcinoma; qRT-PCR, Quantitative real-time polymerase chain reaction. *p < 0.05

Given that *DNASE1L3* inhibits the Wnt/β-catenin pathway and that this pathway is known to modulate immune checkpoints like *PD-L1* in tumor microenvironments [24], we next investigated whether *DNASE1L3* influences *PD-L1* expression in HCC. We studied the correlation between *DNASE1L3* and *PD-L1* expression in HCC cells. qRT-PCR research revealed that *DNASE1L3* overexpression dramatically decreased *PD-L1* mRNA levels in HepG2 cells, whereas *DNASE1L3* knockdown boosted *PD-L1* mRNA expression in Huh7 cells (Fig. 12A). WB analysis confirmed these findings at the protein level in HepG2 and Huh7 cells (Fig. 12B,C). We investigated the impact of *DNASE1L3* knockdown on *PD-L1* expression in relation to Wnt/β-catenin signaling in order to investigate the possible mechanisms behind this regulation. *DNASE1L3* knockdown markedly raised *PD-L1* mRNA levels in HepG2 and Huh7 cells. Treatment with the Wnt/β-catenin pathway inhibitor XAV939 partially reversed the increased *PD-L1* expression caused by *DNASE1L3* knockdown in both cell lines (Fig. 12D,E). Further analysis showed *DNASE1L3* knockdown enhanced Wnt pathway components (Axin2, c-Myc, β-catenin) alongside *PD-L1* in both cell lines (Fig. 12F,H), effects that were attenuated by XAV939, suggesting *DNASE1L3’*s regulatory role in *PD-L1* expression via Wnt/β-catenin pathway. Quantitative analysis of these WB results confirmed that after *DNASE1L3* knockdown in HepG2 and Huh7 cells, the protein levels of Axin2, c-Myc, β-catenin, and *PD-L1* were all significantly increased (Fig. 12G,I).

### 3.13 Knockdown of DNASE1L3 Induces M2-Like Macrophages to Further Promote the Wnt/***β***-Catenin Signaling Pathway

**Figure 13:**
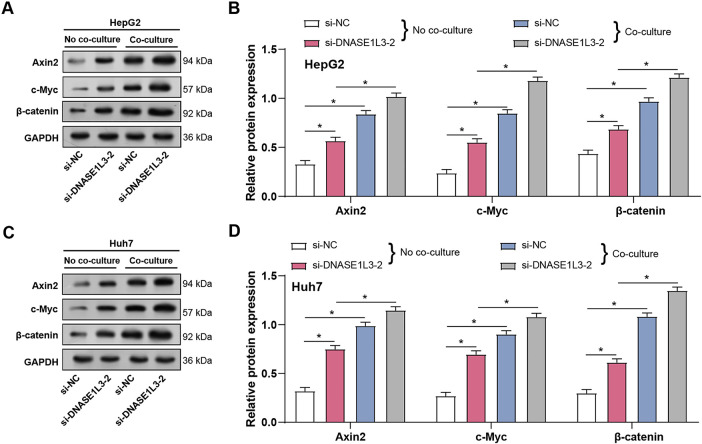
Knockdown of *DNASE1L3* induces M2-like macrophages and further promotes the Wnt/β-catenin pathway. (**A**) WB analysis of Wnt/β-catenin pathway proteins (Axin2, c-Myc, β-catenin) in HepG2 cells transfected with control siRNA (si-NC) or *DNASE1L3* siRNA (si-*DNASE1L3*-2) with or without co-culture of M2-like macrophages. GAPDH was used as a loading control. (**B**) Protein expression levels in HepG2 cells were quantified by WB analysis under the conditions described in (**A**). (**C**) WB analysis of Wnt/β-catenin pathway proteins in Huh7 cells transfected with si-NC or si-*DNASE1L3*-2 with or without co-culture of M2-like macrophages. GAPDH was used as a loading control. (**D**) Protein expression levels in Huh7 cells were quantified by WB analysis under the conditions described in (**C**). *p < 0.05

We investigated the role of *DNASE1L3* in regulating Wnt/β-catenin signaling pathway in HCC cells, both with and without co-culture with M2-like macrophages. As shown in Fig. 13A,B, in HepG2 cells, *DNASE1L3* knockdown led to increased protein expression of Wnt/β-catenin pathway components Axin2, c-Myc, and β-catenmin when there was no co-culture. It is important to note that co-culturing with M2-like macrophages increased this impact. Consistent observations were made in Huh7 cells. Compared to the si-NC group under no co-culture conditions, *DNASE1L3* knockdown resulted in increased protein levels of Axin2, c-Myc, and β-catenin (Fig. 13C,D). This impact was enhanced by co-cultivation with M2-like macrophages, as seen by a significant rise in protein expression as compared to the conditions without co-cultivation. These findings imply that *DNASE1L3* could act in HCC cells as a negative regulator of the Wnt/β-catenin signaling pathway. Furthermore, the enhanced effect observed under co-culture conditions with M2-like macrophages implies that *DNASE1L3* may play a crucial role in regulating Wnt/β-catenin signaling pathway within the tumor microenvironment by interacting with M2-polarized tumor-associated macrophages.

### 3.14 Characterization of Hepatocellular Carcinoma PDOs

**Figure 14:**
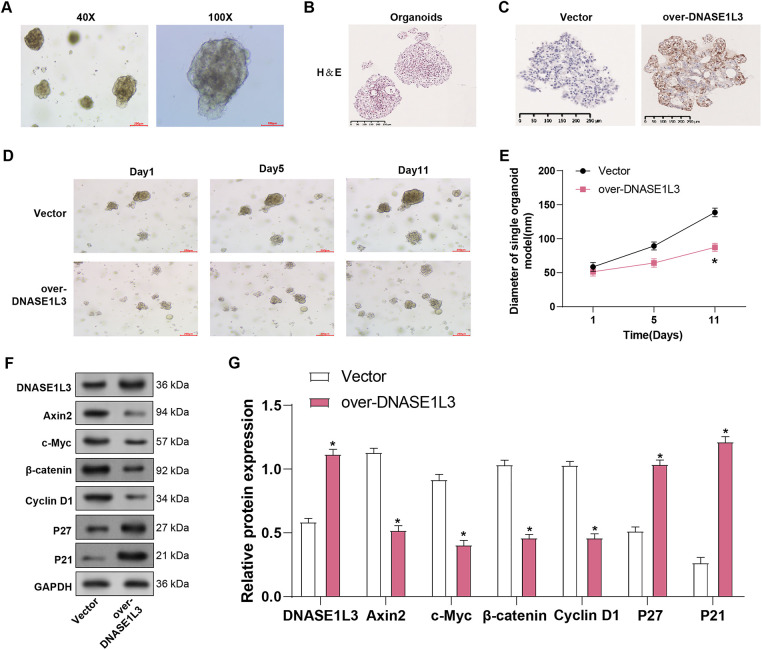
*DNASE1L3* overexpression inhibits liver organoid growth and Wnt/β-catenin signaling pathway. (**A**) Representative brightfield images of HCC PDO imaged using different magnifications (40×, 100×). Scale bar, 100 μm. (**B**) H&E-stained histological sections of HCC PDOs and the corresponding tumors. The cell morphology and arrangement of the original tumors were maintained in the corresponding HCC PDOs. Scale bar, 250 μm. (**C**) Immunohistochemical staining of DNASE1L3 in vector control and DNASE1L3-overexpressing organoids. Scale bar, 250 μm. (**D**,**E**) After 1, 5, and 11 days, the growth of the organoid model overexpressing *DNASE1L3* was evaluated, and the diameter of the organoids was measured. (**F**,**G**) WB analysis of DNASE1L3, Wnt/β-catenin pathway components (Axin2, c-Myc, β-catenin), cell cycle regulator Cyclin D1, and cell cycle inhibitors (p27, p21) in organoid lysates. GAPDH serves as a loading control. HCC, hepatocellular carcinoma; WB, Western blot. *p < 0.05

We investigated the effects of *DNASE1L3* overexpression on liver organoid formation and growth. Liver organoids were successfully generated and observed at different magnifications. Fig. 14A,B shows images of organoids at 40× and 100× magnifications under an optical microscope. Multiple organoids were observed at 40×, and each organoid presented a compact spherical structure with clear boundaries. 100x magnification provided a closer view, showing dense cell arrangement and integrity of the outer layer of the organoid, indicating that the organoids formed a solid histology. Subsequently, paraffin-embedded sections were subjected to histological examination, and *DNASE1L3* expression was increased in organoids overexpressing *DNASE1L3* compared with vector controls (Fig. 14C). To explore the biological function of *DNASE1L3* in HCC organoids, we constructed a human HCC organoid model from resected primary HCC tissue and overexpressed *DNASE1L3*. The growth of the *DNASE1L3* overexpression organoid model was evaluated at 1, 5, and 11 days, and the diameter of the organoid was measured (Fig. 14D). The findings revealed that the development of *DNASE1L3* overexpression organoids was severely suppressed. Overexpression of DNASE1L3 lowered the expression of Wnt/β-catenin pathway proteints (Axin2, c-Myc, β-catenin) and cell cycle regulator Cyclin D1 in HCC organoids (Fig. 14E,F). In contrast, the expression of the cell cycle inhibitors p27 and p21 increased. This shows that *DNASE1L3* suppresses Wnt/β-catenin signaling and cell cycle progression in liver organoids.

## 4 Discussion

HCC is a tumor characterized by a complex immune microenvironment, with immune evasion mechanisms playing a key role in tumor progression [25]. A thorough analysis of the critical role immune checkpoint inhibitors (ICIs) play in the treatment of cancer, especially HCC, was presented by Donne and Lujambio [26]. The FDA approved atezolizumab (anti-PD-L1) plus bevacizumab (anti-VEGF) as the preferred first-line therapy for advanced HCC because of the substantial improvement in overall survival as compared to sorafenib, they stressed. Kotsari et al. [27] highlighted the critical role of the tumor microenvironment in the initiation and progression of HCC, identifying it as one of the three major unresolved challenges in clinical treatment-tumor recurrence, fatal metastasis, and the refractory tumor microenvironment. They provided an in-depth discussion of current therapeutic approaches for HCC, along with potential immune-based treatment strategies aimed at overcoming these persistent obstacles. In this context, DNASE1L3, an enzyme that aids in the clearance of cell debris by cleaving DNA released during cell death, emerges as an important player in maintaining immune tolerance and preventing autoimmunity [28,29]. Recent findings by Wang et al. suggest that *DNASE1L3* expression is associated with improved responses to sorafenib in HCC patients, potentially serving as a biomarker for predicting therapeutic outcomes [30]. Our study, through bioinformatics analysis, identified *DNASE1L3* as a hub gene in HCC and explored its role in HCC cell and macrophage co-culture models. Furthermore, we explored the connection between *DNASE1L3* and the Wnt/β-catenin signaling pathway, offering novel insights into prospective therapies for HCC.

In this study, bioinformatics analysis of the GSE41804 dataset was used to identify DEGs and key modules. These findings were then cross-referenced with DEGs in the TCGA-LIHC dataset, focusing on the overlap between TCGA DEGs-down and turquoise modules to identify overlapping genes. Prognostic analysis of these genes revealed eight significant prognostic markers, including *CPEB3*, *NTF3*, and *CFHR3*. Zhang et al. discovered that CPEB3 suppresses the EMT and metastasis of HCC cells by inhibiting the translation of metadherin (*MTDH*) mRNA [31]. Furthermore, *CPEB3* expression is downregulated in HCC tissues, positioning it as a favorable prognostic marker [32]. Liu et al. discovered that NTF3 is weakly expressed in HCC and correlates favorably with immune cells such as CD4^+^ T cells, mast cells, NK cells, macrophages, as well as B cells, while negatively associating with immune checkpoint markers like PD-L1, TIGIT, and TIM-3 [33]. *CFHR3* expression is modulated by miR-590-3p, which in turn affects the STAT3/p53 signaling pathway to influence the malignant phenotype of HCC. In line with these findings, our analysis of prognostic gene expression across HCC-related datasets revealed consistent downregulation of these genes in tumor samples, indicating that they may act as tumor suppressor genes in HCC.

Additionally, this study demonstrated that *DNASE1L3* was considerably downregulated in HCC and that it was expressed in both normal hepatocytes and HCC cells. This outcome is in line with research by Sun et al., which shown that *DNASE1L3* was downregulated in HCC and that it interacted with *CDK2* to prevent *CDK2*-mediated *E2F1* activation, which in turn delayed the development of the cell cycle and prevented carcinogenesis [34]. Liu and Meng found that *DNASE1L2* was upregulated in breast cancer cells as well as regulated EMT as an oncogene, affecting cell proliferation, invasion and migration, and is expected to become a potential biomarker for breast cancer treatment [35]. A transcription factor called c-Myc controls the course of the cell cycle by encouraging proliferation and preventing differentiation when it is overexpressed [36,37]. In addition, cell cycle-dependent kinase inhibitors P21 and P27 can control the G1 phase, induce cell cycle arrest and inhibit proliferation [38,39]. In our investigation, further overexpression of *DNASE1L3* in HCC cells prevented invasion, migration, and cell division. G1 phase cell cycle arrest resulted from this, which was accompanied by elevated expression of the G1 phase-related proteins P21 and P27. Furthermore, overexpression of *DNASE1L3* resulted in an upregulation of the epithelial marker E-cadherin and a reduction in c-Myc, N-cadherin, and vimentin, suggesting that *DNASE1L3* suppresses EMT in HCC cells. These findings demonstrate *DNASE1L3’*s complex function in HCC, which includes cell cycle control, EMT inhibition, and general tumorigenic property reduction.

HCC co-cultured with macrophages simulates the tumor microenvironment, offering insights into tumor-immune interactions [40]. Macrophages play dual roles, promoting tumor progression via immunosuppression or inhibiting tumor growth through immune activation [41]. Co-cultures enable the study of macrophage polarization, cytokine secretion, and their impact on HCC invasion, proliferation, and metastasis. Understanding how HCC cells influence macrophage phenotype and function aids in identifying therapeutic targets for HCC immunotherapy. Research by Yang and Xing demonstrated that Ligustilide inhibits HCC progression and mitigates Macrophage M2 polarization induced by HCC cells through YAP-mediated interleukin-6 secretion [42]. Yang’s study revealed that the interaction between hepatic tumor cells and macrophages, mediated by Wnt/β-catenin signaling, promotes M2-like macrophage polarization and enhances tumor malignancy in HCC [7]. Additionally, the research of Yin revealed that Emodin suppresses HCC growth by shifting the polarization of macrophages from M2 to M1 through the miR-26a/TGF-β1/Akt axis [43]. Research by Hu et al. reveals that M2 macrophage polarization is induced by exosomal miR-452-5p from HCC cells, hastening HCC progression via TIMP3 targeting [44]. These findings collectively underscore the intricate interplay between HCC cells and macrophages. The Frizzled receptor family members Fzd4, Fzd7, and Fzd9 play an important role in tissue development, cell proliferation, and differentiation by modulating the Wnt signaling pathways [45,46]. In our study, the downregulation of *DNASE1L3* in HCC cells induces polarization towards M2-like macrophages and enhances tumor infiltration. Co-cultivation with THP-1 macrophages revealed varying expression patterns of M1 and M2 markers, modulated by *DNASE1L3* expression levels. Our research also showed that M1 macrophages had lower expression levels of Wnt receptors Fzd4, Fzd7, and Fzd9, but M2 macrophages had much higher expression levels. This suggests that Wnt signaling plays a part in macrophage polarization.

An essential cascade involved in a variety of biological processes, such as tissue homeostasis, embryonic development, and carcinogenesis, is the Wnt/β-catenin signaling pathway [47]. Important components of the Wnt/β-catenin signaling system that control cell division, proliferation, and the advancement of cancer are β-catenin, Axin2, and c-Myc [48,49]. Axin2 regulates negative feedback, β-catenin is a transcription factor, and c-Myc is a transcription factor linked to metabolism and cell division [50,51]. The Wnt/β-catenin pathway is a major target for therapeutic intervention since it is linked to a number of illnesses, including cancer [52]. Pirfenidone, studied by Zou et al., suppresses the proliferation of HCC cells and triggers apoptosis via blocking the Wnt/β-catenin pathway [53]. Moreover, Wang et al. reported that the deubiquitinase USP39, up-regulated in HCC, directly deubiquitinates β-catenin to stabilize it and thereby promotes hepatocellular carcinoma progression; this effect also involves suppressing TRIM26 maturation, collectively enhancing Wnt/β-catenin pathway activity [54]. Additionally, Wang et al. showed that circATF6, downregulated in HCC, suppresses tumor progression by inhibiting CALR-mediated Wnt/β-catenin signaling [55]. Wnt proteins, such as Wnt2, Wnt3, Wnt3a, Wnt4, Wnt10b, and Wnt16, can activate Frizzled receptors and regulate β-catenin-dependent or independent signaling pathways, thereby influencing cancer cell behavior [56]. The long non-coding RNA LNC-POTEM-4 is substantially expressed in HCC, according to recent research by Lin et al. [57]. By upregulating Wnt4 and triggering the Wnt signaling pathway, it serves as a sponge for miR-149-5p, which encourages the malignant development of HCC. According to Zhang et al., CTB-193M12.5 is abundantly expressed in HCC and stimulates the growth of tumors via the WNT10B/Wnt/β-catenin signaling pathway mediated by *NSD1* [58]. Both XAV939 and ICG-001 are small compounds that function as inhibitors of the Wnt/β-catenin signaling system [59]. XAV939 inhibits tankyrase, which stops β-catenin from stabilizing, while ICG-001 blocks the connection between β-catenin and CBP [60,61].

This study found that the expression of downstream genes in the Wnt/β-catenin signaling pathway, including Axin2, β-catenin, and c-Myc, was significantly elevated in M2 macrophages compared to M0 and M1 macrophages, with β-catenin protein levels notably upregulated in M2 macrophages. Further analysis showed that Wnt3, Wnt3a, and Wnt4 were expressed at much higher levels in M2 macrophages than in M0 or M1 macrophages. Additionally, *DNASE1L3* knockdown increased the expression of M2 macrophage markers (CD206 and ARG1) and Wnt/β-catenin pathway components (c-Myc and β-catenin). These findings suggest a potential regulatory link between *DNASE1L3* and macrophage polarization, possibly involving the Wnt/β-catenin signaling pathway. Mechanistically, our results support the possibility that *DNASE1L3* interacts with β-catenin to inhibit Wnt/β-catenin pathway activation by promoting β-catenin ubiquitination and degradation, preventing its nuclear translocation and influencing macrophage polarization. *DNASE1L3* also recruits the β-catenin degradation complex, enhancing its ubiquitination and proteasomal degradation, thereby suppressing β-catenin activation. Collectively, these findings indicate that *DNASE1L3* is likely involved in modulating β-catenin signaling and macrophage polarization, which may contribute to its tumor-suppressive role in HCC.

Importantly, modulation of Wnt signaling pathway has a significant impact on the control of PD-L1, a crucial immunological checkpoint protein implicated in cancer cells evading the immune system [62,63]. The Wnt signaling pathway upregulates *PD-L1* expression, allowing tumor cells to suppress T cell activity and reduce immune responses, thereby promoting immune evasion [64,65]. According to research by Lv et al., GSDMD was linked to a poor prognosis and was elevated in HCC. GSDMD promotes HCC progression by regulating the cGAS pathway and enhancing *PD-L1* expression, thereby promoting immune evasion [66]. Furthermore, the Wnt/β-catenin signaling pathway promotes the binding of the β-catenin/TCF/LEF complex to the PD-L1 gene promoter, in which AKT plays a crucial role, hence upregulating PD-L1 expression, according to research by Du et al. [67]. Inhibition of this pathway can reduce *PD-L1* expression and enhance anti-tumor immune responses, providing a new potential target for tumor immunotherapy. Our research demonstrated that *DNASE1L3* uses the Wnt/β-catenin signaling pathway to negatively control *PD-L1* expression in liver cancer cells. Pharmacological suppression of the Wnt/β-catenin pathway partially attenuated the increase in *PD-L1* expression induced by *DNASE1L3* knockdown, supporting an indirect regulatory relationship. These results are in line with earlier research by Ma et al., which found that overexpressing *PD-L1* increased the migration, invasion, and colony formation of NSCLC cells and activated the MAPK and Wnt/β-catenin signaling pathways, whereas suppressing *PD-L1* decreased these functions [68]. Our results also showed that *DNASE1L3* knockdown amplified the activation of the Wnt/β-catenin signaling pathway by M2 macrophages. This suggests that *DNASE1L3* plays a crucial role in the tumor microenvironment by negatively regulating *PD-L1* expression and macrophage polarization. These findings provide insights into the complex interactions between tumor cells, immune cells, and signaling pathways in HCC progression and immune evasion. Future studies will focus on further investigating the specific mechanisms underlying the regulatory relationship between the Wnt/β-catenin pathway and *PD-L1* in HCC.

PDOs maintain the pathological and molecular characteristics of cancer, offering high experimental accessibility and lower costs compared to animal models [69]. PDOs are superior to cell lines in representing the natural state of tumors and may serve as suitable models for potential therapeutic agents. Jia et al. demonstrated that PDO is a valuable tool for assessing the therapeutic potential of oroxylin A for HCC [70]. Oroxylin A, a novel transketolase inhibitor, effectively suppresses the non-oxidative pentose phosphate pathway (PPP), leading to the inhibition of HCC growth in both murine models and PDOs. Similarly, Li et al. showed that Omacetaxine, an FDA-approved treatment for chronic myelogenous leukemia (CML), exhibits efficacy in HCC PDOs at low concentrations [71]. Mechanistic investigations revealed that omacetaxine exerts its effects by inhibiting global protein synthesis, targeting key oncogenes such as PLK1. This emphasizes the importance of PDO in HCC drug discovery and suggests omataxine as a promising treatment option for HCC patients. The above is the same as our experimental results. Our study found that HCC PDO exhibited morphological similarity to the original tumor and maintained histological characteristics. Overexpression of *DNASE1L3* in HCC organoids reduced growth and changed critical signaling pathways. Organoids overexpressing *DNASE1L3* became smaller and slower within 11 days. *DNASE1L3* overexpression decreased Wnt/β-catenin pathway components and promoters, while increased cell cycle inhibitors. These findings imply that *DNASE1L3* suppresses HCC growth by blocking the Wnt/β-catenin pathway and cell cycle progression in 3D organoids.

Despite the comprehensive analyses performed in this study, several limitations should be acknowledged. First, the sample size for clinical specimens was very small, which may limit the generalizability of our findings. Larger cohorts or validation using public datasets are needed to support the clinical relevance of *DNASE1L3* in HCC. Second, some key controls were missing, such as rescue experiments for *DNASE1L3* knockdown or overexpression in organoid models, which could strengthen the mechanistic conclusions. Third, while *in vitro* and organoid models provide valuable insights, *in vivo* validation is still lacking, and the observed effects may not fully represent the complexity of HCC progression in patients. Future studies should address these limitations to further confirm the tumor-suppressive role of *DNASE1L3*.

## 5 Conclusion

We established that *DNASE1L3* is important in reducing HCC development using a multidimensional strategy that included bioinformatics research, *in vitro* tests, and organoid models. Specifically, *DNASE1L3* inhibited HCC cell malignant behavior while inducing G1 cell cycle arrest and regulating epithelial-mesenchymal transition markers. *DNASE1L3* suppresses the Wnt/β-catenin signaling cascade by binding with and degrading β-catenin. This mechanism extends to regulating PD-L1 expression and influencing macrophage polarization in the tumor microenvironment, favoring an anti-tumor M1 phenotype. The tumor suppressive role of *DNASE1L3* was further validated in HCC organoids, reinforcing its potential as a therapeutic target. Together, these findings emphasize the diverse roles of *DNASE1L3* in HCC, encompassing its direct impact on cancer cells and regulation of the tumor microenvironment, pointing to its potential as a therapeutic target for modulating tumor progression and the surrounding environment.

## Data Availability

The data that support the findings of this study are available from the Corresponding Authors, [Qianqian Cai, Fei Fan], upon reasonable request.
